# Association Between Composite Dietary Antioxidant Index and Depressive Symptoms in Breast Cancer Patients: The Role of Oxidative Stress Biomarkers in a Cross-Sectional Study

**DOI:** 10.3390/nu18142230

**Published:** 2026-07-09

**Authors:** Ran Wang, Jianyun He, Lan Cheng, Zhenzhen Huang, Xinyi Miao, Xinxin Cheng, Yuting Wang, Xiaoxia Lin, Shufang Xia

**Affiliations:** Wuxi School of Medicine, Jiangnan University, Wuxi 214122, China; wangran@stu.jiangnan.edu.cn (R.W.); hejianyun@stu.jiangnan.edu.cn (J.H.); chenglan@stu.jiangnan.edu.cn (L.C.); huangzhenzhen@stu.jiangnan.edu.cn (Z.H.); miaoxinyi@stu.jiangnan.edu.cn (X.M.); chengxinxin@stu.jiangnan.edu.cn (X.C.); wangyuting@stu.jiangnan.edu.cn (Y.W.); linxiaoxia@stu.jiangnan.edu.cn (X.L.)

**Keywords:** composite dietary antioxidant index, breast cancer, depressive symptoms, oxidative stress, antioxidants

## Abstract

**Background**: Depressive symptoms (DepS) are prevalent among breast cancer patients and are associated with poor treatment outcomes and prognosis. Oxidative stress has been implicated in the pathophysiology of depression, and dietary antioxidants may be associated with oxidative balance. This study aimed to evaluate the association between the composite dietary antioxidant index (CDAI) and DepS, and to explore the statistical relationships between CDAI, oxidative stress biomarkers, and DepS. **Methods**: In this cross-sectional study, 302 breast cancer patients were enrolled, of whom 113 provided blood samples for the assessment of plasma oxidative stress biomarkers. Dietary intake was assessed using 3-day 24 h dietary recalls. The CDAI was calculated based on the intake of six dietary antioxidants, including vitamins A, C, and E, zinc, selenium, and manganese. DepS were defined as a score of ≥8 on the Hospital Anxiety and Depression Scale—Depression subscale (HADS–D). **Results**: A total of 102 patients (33.77%) exhibited DepS, and these patients had significantly lower CDAI scores and reduced plasma levels of superoxide dismutase (SOD) and glutathione (GSH), compared with those without DepS (all *p* < 0.05). CDAI and dietary zinc intake were non-linearly associated with DepS (*p* for non-linearity <0.05). Vitamin C intake was inversely associated with DepS (OR = 0.989; 95% CI: 0.984, 0.993; *p* < 0.001). CDAI scores were positively associated with SOD and GSH levels (both *p* < 0.001), while SOD and GSH were inversely associated with DepS (SOD: β = −0.610; GSH: β = −0.900; both *p* < 0.001). In exploratory mediation analysis, GSH and SOD statistically accounted for part of the association between CDAI and DepS (ACME = −0.192 and −0.228, respectively; all *p* < 0.01). **Conclusions**: Higher dietary antioxidant intake, as reflected by CDAI, is associated with lower DepS in breast cancer patients, and oxidative stress biomarkers may be statistically involved in this association.

## 1. Introduction

Breast cancer remains a prominent global health challenge, ranking second in incidence and fifth in cancer-related mortality among female malignancies in China [1]. Beyond the substantial physical burden of the disease, breast cancer is frequently accompanied by psychological distress, particularly depressive symptoms (DepS), which may occur across the disease trajectory, including at diagnosis, during treatment, and in the survivorship phase. The prevalence of DepS in breast cancer patients varies widely, ranging from approximately 8% to over 80%, with recent estimates suggesting rates as high as 44.1% [2,3]. Our previous study also reported a prevalence of 27.2% [4]. Importantly, DepS are often under-recognized in clinical practice due to overlap with cancer-related somatic symptoms and adverse effects of chemotherapy [5]. Moreover, depression has been reported to be associated with poor breast cancer outcomes, including increased risks of recurrence and mortality, with pooled hazard ratios ranging from 1.17 to 1.34 [6]. These findings underscore the significant clinical burden of DepS in breast cancer patients.

Despite the significant burden of DepS, effective strategies for alleviating these symptoms in this population remain limited. As a modifiable lifestyle factor, diet has gained increasing attention for its potential role in mental health. Epidemiological evidence suggests that dietary patterns rich in vegetables and fruits are associated with a reduced risk of depression [7], whereas diets high in processed foods, refined grains, sugar-sweetened beverages, and red meat are linked to an increased risk of depression [8]. One potential biological mechanism underlying these associations is oxidative stress. An imbalance between reactive oxygen species (ROS) and antioxidant defenses can lead to cellular damage and neurobiological alterations, which are thought to be involved in the pathophysiology of DepS [9]. In fact, biomarkers of oxidative stress, such as malondialdehyde (MDA) and antioxidant enzymes including superoxide dismutase (SOD), catalase (CAT), and glutathione peroxidase (GSH-Px), have been shown to be altered in individuals with depression. Notably, antidepressant treatments have been shown to modulate oxidative stress biomarkers. For example, escitalopram significantly reduced plasma SOD, CAT and MDA levels in patients with major depressive disorder (MDD) [10]. These findings suggest that oxidative stress may be one of the biological processes involved in the association between dietary antioxidant intake and DepS.

At the micronutrient level, dietary antioxidant components include both non-enzymatic antioxidants and antioxidant-related trace elements, which contribute to the maintenance of systemic redox homeostasis and protection against oxidative stress [11]. Non-enzymatic antioxidants, including vitamins A, C, and E, directly scavenge ROS and inhibit lipid peroxidation, thereby supporting cellular redox balance [12]. Specifically, vitamin C efficiently neutralizes free radicals in aqueous compartments, while lipophilic vitamin E helps protect cell membranes from oxidative damage by limiting lipid peroxidation [12,13]. Following intestinal absorption, antioxidant nutrients are distributed via the circulation and may influence systemic oxidative status [14]. Emerging evidence suggests that antioxidant vitamins may help maintain cellular redox balance and modulate oxidative stress-related biological processes, including regulation of cellular oxidative balance and inflammatory signaling pathways [15]. Reduced circulating levels of antioxidant vitamins have also been observed in breast cancer patients, potentially reflecting increased oxidative burden associated with the disease [16]. Several antioxidant-related trace elements, including zinc, selenium, and manganese, contribute to endogenous antioxidant defense by serving as cofactors for antioxidant enzymes and supporting the maintenance of cellular redox homeostasis [17]. Zinc and selenium are essential components of antioxidant defense systems, and deficiencies in these minerals have been associated with impaired antioxidant capacity and increased susceptibility to oxidative stress [18]. Manganese serves as an essential cofactor for mitochondrial superoxide dismutase (MnSOD), a key antioxidant enzyme that protects cells against oxidative damage by catalyzing the dismutation of superoxide radicals [19]. Collectively, impaired antioxidant defenses may contribute to systemic oxidative imbalance, which has been implicated in DepS in clinical populations [20].

To integrate the overall antioxidant capacity of diet, the Composite Dietary Antioxidant Index (CDAI) was developed as a comprehensive measure of dietary antioxidant exposure [21]. Compared with studies focusing on individual nutrients, CDAI may better reflect the combined antioxidant potential of the diet. Previous studies have reported an inverse association between CDAI and DepS in middle-aged and older adults, as well as in overweight and obese adults [22,23]. However, evidence regarding this association in breast cancer patients undergoing chemotherapy remains limited, which highlights an important research gap.

Therefore, we hypothesized that higher dietary antioxidant intake, as reflected by a higher CDAI, is associated with lower DepS in breast cancer patients undergoing chemotherapy. Furthermore, we aimed to explore whether oxidative stress biomarkers, including MDA, SOD, CAT, GSH and GSH-Px, may statistically account for part of the association between CDAI and DepS, thereby providing exploratory insights into diet-oxidative stress-DepS correlations in this population.

## 2. Materials and Methods

### 2.1. Participants and Study Design

This cross-sectional study was conducted at the Affiliated Hospital of Jiangnan University from December 2025 to April 2026. The inclusion criteria included: female patients with histopathologically confirmed breast cancer, without distant metastasis or local progression, hospitalization for postoperative chemotherapy; aged ≥18 years; normal cognitive function and reading ability; and willing to participate in the study and provided written informed consent. The exclusion criteria were: a history of or current physician-diagnosed psychiatric disorders; had other concurrent malignancies or terminal diseases; diagnosed chronic diseases that could affect dietary intake, nutrient absorption; receiving targeted therapy or radiotherapy; use of sedatives, anxiolytics, antidepressants, or other medications potentially affecting mood or emotional state within the past three weeks; use of dietary supplements, including but not limited to vitamins C, and E, selenium, manganese, fish oil, or other supplements potentially affecting oxidative stress within the past three months, and incomplete questionnaire or clinical data. The study was approved by the Medical Ethics Committee of the Affiliated Hospital of Jiangnan University on 26 December 2025 (Approval No. LS2025441) and registered at the Chinese Clinical Trial Registry (ChiCTR2600120727). Participant recruitment commenced on 30 December 2025. All procedures were in accordance with the principles of the Declaration of Helsinki (1989), and all participants provided written informed consent.

### 2.2. Sample Size

The prevalence of DepS in Chinese breast cancer patients undergoing chemotherapy, as assessed by the HADS-D score, was reported to be 44.1% [3]. Based on this prevalence, the sample size was estimated using the following formula: $n\;=\;\frac{Z_{\alpha /2}^{2}\cdot p(1\;-p)}{\delta^{2}}$, where *α* = 0.05, confidence level = 1 − *α* = 95%, *Z_α_*_/2_ = 1.96, *p* = 0.441, and *δ* = 0.06 (permissible error of 6%), chosen to balance statistical precision and feasibility [24]. The calculated sample size was 296 participants. For mediation analysis, following the guidelines of Fritz et al. [25], the required sample size was estimated using a medium effect size a = b = 0.39, which indicated that 71 participants would be sufficient for 80% power at *α* = 0.05. Therefore, the larger sample size derived from the prevalence estimation was adopted for this study to ensure statistical power and generalizability.

### 2.3. Assessment of Depressive Symptoms

DepS were assessed using the Hospital Anxiety and Depression Scale—Depression subscale (HADS-D), a widely validated 14-item self-report instrument commonly used in oncology settings [26]. The HADS comprises two 7-item subscales measuring anxiety and depression, respectively [26]. Each item is scored on a scale from 0 to 3, with a total depression score ranging from 0 to 21. According to established cutoffs, a score of 7 or below indicates the absence of DepS, while a score of 8 or higher suggests the presence of DepS [26].

### 2.4. Assessment of Dietary Intake and Calculation of CDAI

Dietary intake was assessed using three non-consecutive 24 h dietary recalls. The first recall was conducted upon hospital admission through face-to-face interviews, during which participants recorded all foods consumed on the previous day. The remaining two recalls were conducted after discharge, one on a weekday and one on a weekend day, via WeChat or telephone once chemotherapy-related gastrointestinal symptoms had resolved. Information on food types, portion sizes, and cooking methods was collected with the aid of food models and atlases, and caregivers assisted when necessary. Incomplete dietary information was verified through follow-up contact to ensure data accuracy. Dietary data were analyzed using the Nutrition Calculator (version 2.8.3.0, Beijing, China) based on the Sixth Edition of the China Food Composition Tables to calculate average daily nutrient intake. *n*-3 polyunsaturated fatty acids (*n*-3 PUFAs) were additionally derived from the same food composition database.

In this study, the CDAI was constructed using six dietary antioxidant nutrients, including manganese, selenium, zinc, and vitamins A, C, and E [22,23]. The intake of each nutrient was standardized by subtracting the corresponding mean intake and dividing by its standard deviation, both derived from the study population for each nutrient. The standardized values of the six nutrients were then summed to obtain the CDAI score for each participant, as shown in the following formula:$\;\mathrm{CDAI}\;=\;\sum_{i=1}^{n=6}\frac{\mathrm{Individual}\;\mathrm{Intake}\;-\mathrm{Mean}}{\mathrm{SD}}$. Because no standardized energy-adjusted CDAI calculation method has been established in the literature, total energy intake was included as a covariate in all multivariable analyses. Higher CDAI scores indicate greater overall dietary antioxidant capacity.

### 2.5. Oxidative Stress Biomarkers Determination

Prior to the initiation of chemotherapy, due to participant willingness, only 113 participants provided fasting venous blood samples. Blood samples were processed within 30 min after collection. Samples were centrifuged at 3500 rpm for 10 min to obtain plasma, which was immediately aliquoted and stored at −80 °C until analysis. The biomarkers measured included SOD, GSH-Px, GSH, CAT, and MDA, using assay kits from Nanjing Jiancheng Bioengineering Institute (Nanjing, China). Specifically, SOD activity was measured using the xanthine–xanthine oxidase system coupled with WST-1, based on the inhibition of WST-1 reduction to a water-soluble formazan dye (Cat. No. A001-3). GSH-Px activity was measured based on the enzymatic reduction of hydrogen peroxide using GSH as substrate, with activity calculated from the rate of GSH consumption (Cat. No. A005-1). GSH concentration was determined using the 5,5′-dithiobis-(2-nitrobenzoic acid) (DTNB) colorimetric method (Cat. No. A006-2-1). CAT activity was determined based on the decomposition of hydrogen peroxide, with residual H_2_O_2_ reacting with ammonium molybdate to form a yellow complex measured spectrophotometrically (Cat. No. A007-1-1). MDA concentration was measured using the thiobarbituric acid (TBA) method based on the formation of an MDA–TBA adduct with maximal absorbance at 532 nm (Cat. No. A003-1). All measurements were performed in triplicate for each sample, strictly following the manufacturer’s protocols.

### 2.6. Other Variables

Sleep disturbance (SD) was evaluated using the Chinese version of the Pittsburgh Sleep Quality Index (PSQI), with a total score ≥ 8 indicating the presence of SD, and a score < 8 indicating no SD [27]. Physical activity was assessed using the short form of the International Physical Activity Questionnaire (IPAQ-SF), from which the metabolic equivalent of task (MET) was calculated. Based on MET values, physical activity was classified into three categories: low <600 MET·min/week, moderate (600–1500 MET·min/week), and high >1500 MET·min/week [28]. Pain intensity was measured using the Visual Analogue Scale (VAS) [29].

### 2.7. Statistical Analysis

Statistical analysis was performed using SPSS 26.0 (IBM SPSS Inc., Chicago, IL, USA) and R 4.2.2 (R Foundation for Statistical Computing, Vienna, Austria). Continuous variables were tested for normality using the Kolmogorov–Smirnov test. Normally distributed data were expressed as mean ± standard deviation and compared using independent samples *t*-tests, whereas non-normally distributed data were expressed as median (25th, 75th percentiles) and compared using the Mann–Whitney U test. Categorical variables were expressed as frequencies (n) and percentages (%) and compared using Chi-square or continuity-corrected Chi-square tests. For descriptive analyses and logistic regression models, DepS was defined as HADS-D ≥ 8. For analyses evaluating the associations between oxidative stress biomarkers and DepS, the continuous HADS-D score was used as the dependent variable in linear regression models. Logistic regression was performed to assess the associations between CDAI, its dietary components, and DepS, with odds ratios (ORs) and 95% confidence intervals (CIs) reported. To explore potential non-linear relationships, restricted cubic spline (RCS) regression was performed with four knots at the 5th, 35th, 65th, and 95th percentiles of the exposure distribution. The median value of the exposure variable was used as the reference value (OR = 1.00). Likelihood ratio tests were applied to compare linear and RCS models. The Akaike Information Criterion (AIC) and Bayesian Information Criterion (BIC) were used to evaluate model fit. Multivariable linear regression was conducted to assess the associations between CDAI and plasma oxidative stress biomarkers, as well as between oxidative stress biomarkers and continuous HADS-D score, adjusting for potential confounders including age, BMI, education level, family monthly income, cancer stage, chemotherapy cycle, PSQI score and total energy intake. Given previous evidence linking *n*-3 PUFAs and folate intake to DepS [30,31], additional multivariable logistic regression analyses were performed to examine their associations with DepS. Biomarkers that were right-skewed were log-transformed prior to analysis. To correct for potential selection bias in the biomarker subgroup, an inverse probability weighting (IPW) sensitivity analysis was conducted. Propensity scores representing the probability of providing a blood sample were estimated using a multivariable logistic regression model including CDAI, age, BMI, education level, family monthly income, chemotherapy cycle, cancer stage, PSQI score, and total energy intake. Stabilized inverse probability weights were derived from these propensity scores and incorporated into the weighted regression models to evaluate the robustness of the primary findings. Trend tests were performed by assigning the median value of each CDAI tertile as a continuous variable. Given the cross-sectional design, mediation analyses were interpreted as exploratory statistical decompositions of associations rather than evidence of causal pathways. Mediation analysis was performed using the R mediation package (v4.5.0) to estimate the average causal mediation effect (ACME), average direct effect (ADE), and average total effect (ATE) with 5000 bootstrap replicates. Model assumptions, including multicollinearity (VIF < 5), linearity in the logit, residual normality, homoscedasticity, and influential points (Cook’s distance and leverage), were examined to ensure model robustness. The hypothesized association framework, exploring how the association between CDAI and DepS is statistically accounted for by oxidative stress biomarkers SOD, CAT, MDA, GSH, GSH-Px, is presented in Figure S1.

## 3. Results

### 3.1. Participants’ Characteristics

As shown in Table 1, among the 302 breast cancer patients included in this study, 102 participants (33.77%) were classified as having DepS, as indicated by HADS-D scores ≥ 8, while 200 participants (66.23%) were classified as not having DepS. Statistically significant differences in the characteristics of the two groups were observed in terms of education level (*p* = 0.041), family monthly income (*p* = 0.017), and PSQI scores (*p* = 0.003).

### 3.2. Nutrient Intake in Patients with and Without Depressive Symptoms

The differences in CDAI scores and nutrient intakes between breast cancer patients with and without DepS are shown in Table 2. Compared with the patients without DepS, the participants with DepS had significantly lower CDAI scores (*p* < 0.001). In addition, vitamin C, manganese, *n*-3 PUFAs and folate intakes were significantly lower in the participants with DepS (*p* < 0.05), while the intake of vitamin A, vitamin E, zinc, and selenium was also lower, but the differences were not statistically significant (*p* > 0.05).

### 3.3. Association of CDAI (and Its Components) with Depressive Symptoms

#### 3.3.1. Assessment of the Linearity Between CDAI (and Its Components) and Depressive Symptoms

RCS models were used to examine the potential non-linear associations between CDAI and DepS. As shown in Table 3, CDAI demonstrated a significant non-linear association with DepS (*p* for non-linearity = 0.024). Zinc intake also showed a strong non-linear relationship (*p* < 0.001), with a lower AIC value, indicating a better fit for the non-linear model. In contrast, vitamin A, vitamin C, vitamin E, manganese, and selenium intakes did not show significant evidence of non-linearity (all *p* > 0.05), supporting the hypothesis of linear associations with DepS.

#### 3.3.2. Non-Linear Associations of CDAI and Zinc with Depressive Symptoms

**Figure 1:**
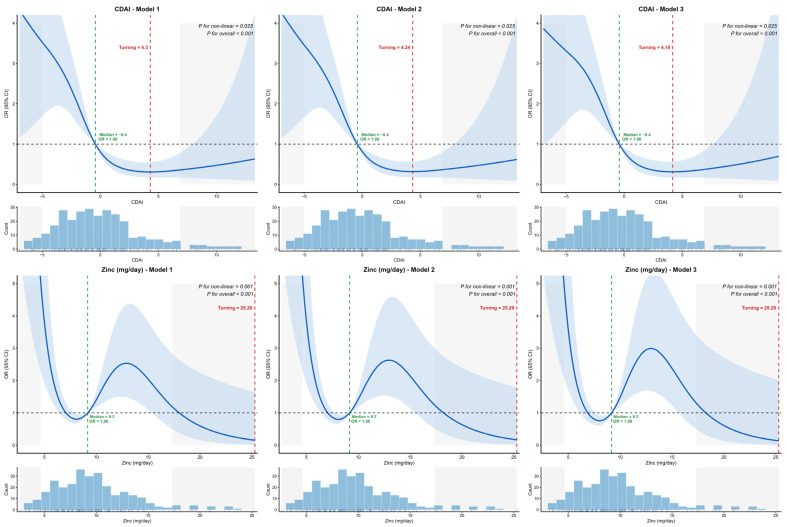
Restricted cubic spline analysis of the associations between CDAI (upper panels) and zinc intake (lower panels) with the odds of depressive symptoms across three models: Model 1 (unadjusted), Model 2 (adjusted for age and BMI), and Model 3 (fully adjusted for age, BMI, education level, family monthly income, cancer stage, chemotherapy cycle, PSQI scores and total energy intake). Lower subpanels show the observed frequency distribution of participants across exposure levels. Grey shaded areas represent regions below the 5th percentile and above the 95th percentile, where data were sparse and estimates should be interpreted cautiously. Green dashed lines indicate the reference value (median exposure level; OR = 1.00), and red dashed lines indicate the estimated turning point of the association.

As shown in Figure 1, RCS analyses revealed significant non-linear associations of CDAI and zinc intake with the odds of DepS across all three models. For CDAI, the odds of DepS decreased with increasing CDAI, reaching the lowest level near the median value (−0.4), followed by a potential flattening or mild upward tendency at higher levels, although the estimates were less precise in the upper distribution range (*p* for non-linearity = 0.025). For zinc intake, a decreasing trend in the odds of DepS was observed with increasing intake, reaching the lowest point near the median intake level, followed by a possible non-linear fluctuation at higher intake levels (*p* for non-linearity <0.001). However, confidence intervals widened substantially at higher zinc intake levels, suggesting increased uncertainty in this range.

#### 3.3.3. Linear Associations of CDAI Components with Depressive Symptoms

In the fully adjusted model (Model 3, Table 4), vitamin C intake was significantly inversely associated with DepS (OR = 0.989; 95% CI: 0.984, 0.993; *p* < 0.001), whereas vitamin A (OR = 1.000; 95% CI: 0.999, 1.001; *p* = 0.963), vitamin E (OR = 0.991; 95% CI: 0.953, 1.028; *p* = 0.642), selenium (OR = 1.000; 95% CI: 0.993, 1.007; *p* = 0.915), and manganese (OR = 0.943; 95% CI: 0.844, 1.005; *p* = 0.201) were not significantly associated with DepS. Results from Model 1 and Model 2 are presented in Supplementary Table S1.

### 3.4. Associations of Dietary n-3 Polyunsaturated Fatty Acids and Folate with Depressive Symptoms

To further explore the potential associations between individual nutrient intakes and DepS, both linear and non-linear models were evaluated for dietary *n*-3 PUFAs and folate intake. Likelihood ratio tests indicated no significant evidence of non-linearity for either *n*-3 PUFAs intake or folate intake (both *p* for non-linearity >0.05, Table S2). Therefore, logistic regression models were adopted. After adjusting for age, BMI, education level, family monthly income, cancer stage, chemotherapy cycle, PSQI score, and total energy intake, neither dietary *n*-3 PUFAs intake nor folate intake was significantly associated with DepS (all *p* > 0.05; Table S3).

### 3.5. Plasma Oxidative Stress Biomarkers in Participants with and Without Depressive Symptoms

To assess potential non-response bias, we compared the demographic characteristics, clinical variables, and CDAI scores between participants who provided blood samples for oxidative stress biomarker assessment (*n* = 113) and those who did not (*n* = 189). As shown in Table S4, no significant differences were observed between the two groups on most variables (all *p* > 0.05). However, participants who provided blood samples had slightly lower PSQI scores (*p* < 0.05). To minimize the potential influence of this difference, PSQI was included as a covariate in all adjusted analyses. In addition, IPW sensitivity analyses were conducted and yielded results that were directionally and statistically consistent with the primary analyses (Table S5), suggesting that potential selection bias is unlikely to have materially influenced the findings.

**Figure 2:**
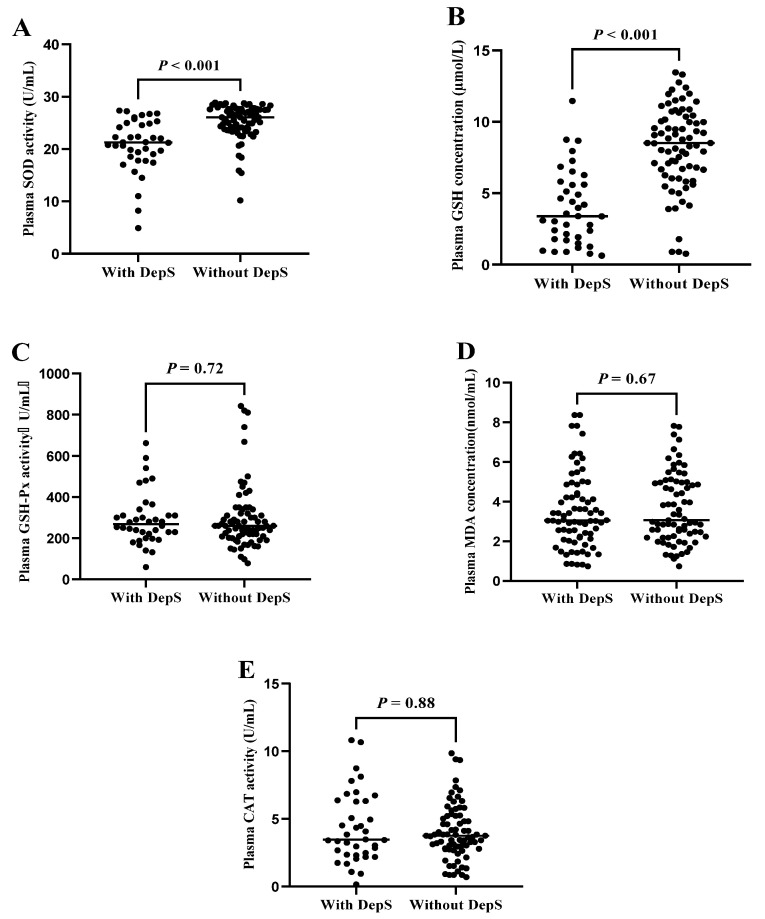
Plasma oxidative stress biomarkers in participants with and without depressive symptoms. (**A**) SOD activity; (**B**) GSH level; (**C**) GSH-Px activity; (**D**) MDA level; (**E**) CAT activity. Data are shown as median (25th, 75th percentiles). Mann–Whitney U test was used. DepS, depressive symptoms; SOD, superoxide dismutase; GSH, glutathione; GSH-Px, glutathione peroxidase; MDA, malondialdehyde; CAT, catalase.

Plasma SOD activity and GSH concentration were significantly lower among participants with DepS than among those without DepS (both *p* < 0.001; Figure 2A,B). In contrast, no significant differences were observed in plasma GSH-Px activity, MDA levels, or CAT activity between the two groups (*p* = 0.72, 0.67, and 0.88, respectively; Figure 2C–E).

### 3.6. Association Between CDAI and Plasma Oxidative Stress Biomarkers

As shown in Table 5, multivariable linear regression analysis revealed that higher CDAI scores were significantly associated with higher levels of SOD and GSH (both *p* < 0.001). Model diagnostics results are presented in Table S6. Therefore, SOD and GSH were retained for subsequent mediation analyses.

### 3.7. Association Between Plasma Oxidative Stress Biomarkers and Depressive Symptoms

After adjusting for covariates, plasma SOD and GSH levels were significantly negatively associated with HADS-D score in breast cancer patients (Table 6). Specifically, SOD (β = −0.610; 95% CI: −0.732, −0.489; *p* < 0.001) and GSH levels (β = −0.900; 95% CI: −1.045, −0.756; *p* < 0.001) were both inversely associated with HADS-D score. Model diagnostics results are presented in Table S7.

### 3.8. Exploratory Mediation Analysis of Oxidative Stress Biomarkers

As shown in Table 7, SOD and GSH statistically accounted for part of the association between CDAI and HADS-D score (Table 7). The total effect (ATE) of CDAI on HADS-D score was −0.918 (95% CI: −1.081, −0.809; *p* < 0.001) for both mediators. SOD statistically accounted for 14.1% of the observed association (ACME = −0.129, 95% CI: −0.228, −0.050; *p* < 0.001), while GSH statistically accounted for 20.9% of the observed association (ACME = −0.192, 95% CI: −0.338, −0.103; *p* < 0.001).

## 4. Discussion

This study found that lower CDAI scores were associated with a higher likelihood of DepS in breast cancer patients. Specifically, patients with DepS had lower intakes of vitamin C and manganese, with vitamin C showing a significant inverse relationship with DepS, while zinc intake exhibited a significant non-linear association with DepS. In addition, plasma SOD and GSH levels were significantly lower among patients with DepS, and both biomarkers statistically accounted for part of the association between CDAI and HADS-D score. These findings suggest that dietary antioxidant intake and oxidative stress biomarkers may be associated with DepS among breast cancer patients.

DepS is highly prevalent among breast cancer patients, with 33.77% of participants in our study reporting DepS. This prevalence was slightly lower than that reported in some previous studies [3], which may partly reflect the use of the HADS-D scale, a tool designed to minimize the influence of cancer-related somatic symptoms on depression assessment [32,33]. Another possible reason is the exclusion of patients with a history of mental illness or those currently diagnosed with psychiatric disorders. Previous studies have consistently shown that cancer patients with a history of mental illness have a significantly increased risk of depression [34]. In this study, many participants had relatively low educational attainment (junior high school or below), and a large proportion had low household incomes. Generally, among Chinese adults, higher educational attainment is associated with lower levels of DepS, which may be related to healthier lifestyles, enhanced health literacy, and improved coping capacity [35]. Conversely, lower household income is typically associated with higher levels of DepS, potentially due to economic stress, limited access to resources, and perceived social disadvantage [36]. Additionally, we also observed a significant difference in PSQI scores between patients with and without DepS. Previous studies have reported that sleep disturbances and DepS frequently coexist in breast cancer patients undergoing chemotherapy and may mutually reinforce one another [37]. Therefore, patients with lower educational attainment, lower socioeconomic status, and poorer sleep quality may warrant closer psychological assessment and monitoring during chemotherapy.

There is growing evidence that diet quality plays an important role in DepS among breast cancer patients [4]. In this study, we found a significant non-linear association between CDAI and DepS. Previous studies have similarly reported that higher CDAI was associated with a lower burden of DepS in both general and clinical populations, including U.S. adults from NHANES 2007–2018, and individuals with overweight/obesity or prediabetes [23,38,39]. However, estimates at the extreme ends of the CDAI distribution should be interpreted cautiously because of the smaller number of observations and wider confidence intervals in these regions. Together, these findings suggest that dietary antioxidant intake may be associated with DepS among breast cancer patients and provide a basis for further investigation of oxidative stress-related pathways underlying this association.

Although the mechanisms underlying the association between CDAI and depression are not fully elucidated, oxidative stress is likely a key factor. Oxidative stress arises from an imbalance between pro-oxidant and antioxidant systems, leading to excessive production of ROS, which can induce cellular damage and neurobiological alterations that have been implicated in the pathophysiology of depression [40]. The brain is particularly vulnerable to oxidative stress due to its high oxygen consumption, abundant lipid content, and relatively limited antioxidant defenses. Emerging evidence suggests that depression is associated with structural and functional alterations in the brain, including reduced volume of the frontal cortex and hippocampus [41]. Oxidative stress has been proposed as a key contributor to these neurological changes, as supported by both preclinical and clinical studies [42,43]. Consistently, a meta-analysis of 23 studies demonstrated that DepS are associated with elevated oxidative stress and reduced antioxidant capacity [44]. Diet has been increasingly recognized as a critical modulator of oxidative stress, influencing the balance between oxidative damage and antioxidant defense systems. These systems include both enzymatic antioxidants, such as GSH-Px, CAT, and SOD, and non-enzymatic antioxidants, such as GSH. A cross-sectional study reported that higher dietary total antioxidant capacity (DTAC) was associated with lower serum MDA levels and reduced DepS [45]. Moreover, the Mediterranean diet, rich in plant-based foods and olive oil, has been shown to enhance antioxidant defenses by increasing SOD activity and reducing MDA levels [46]. An intervention study in athletes also showed that even short-term antioxidant-rich diets (just two weeks) can rapidly increase SOD activity and overall antioxidant capacity [47], suggesting that dietary factors may be associated with oxidative stress regulation and antioxidant defenses. In the present study, SOD and GSH statistically accounted for part of the association between CDAI and HADS-D score, whereas no significant difference was observed in GSH-Px, CAT and MDA levels between patients with and without DepS. However, given the cross-sectional design, these findings should be interpreted as statistical associations rather than evidence of temporal or causal pathways linking dietary antioxidant intake, oxidative stress biomarkers, and DepS. Therefore, the observed mediation effects should be regarded as exploratory and hypothesis-generating, and future longitudinal studies are needed to clarify these relationships. Notably, evidence regarding oxidative stress biomarkers in depression is inconsistent, with some studies reporting increased and others reporting decreased activities of SOD, GSH-Px, and CAT [48,49,50], while GSH appears to be more consistently reduced [51]. For instance, some studies have shown reduced GSH-Px activity in patients with depression, with levels negatively correlated with symptom severity [52], while others have found no significant differences compared to healthy controls [53]. Similar inconsistencies have been reported for CAT and SOD [48,54]. Additionally, MDA, a marker of lipid peroxidation, has been widely linked to the development of MDD [55]. The lack of significant differences in certain biomarkers in our study may reflect the complex and dynamic nature of the antioxidant defense system. One possible explanation is that different oxidative stress biomarkers may respond differently to systemic oxidative status, although the underlying mechanisms remain unclear. SOD and GSH may be more sensitive and immediate indicators of redox imbalance, particularly in the early stages of oxidative stress, while enzymes like GSH-Px and CAT may be subject to compensatory regulation [56], potentially masking observable group differences. In contrast, MDA, as a final product of lipid peroxidation, serves as a more distal marker of oxidative damage and can reflect the cumulative effects of prolonged oxidative stress. In addition, heterogeneity within the breast cancer population, including factors like cancer treatment and inflammatory burden, may influence antioxidant responses. Furthermore, it is also possible that different oxidative stress markers reflect distinct biological pathways, and not all are equally involved in the link between CDAI and DepS. Future studies could consider incorporating a broader range of oxidative stress markers to provide a more comprehensive assessment of their role in the association between dietary antioxidant capacity and DepS.

At the level of individual dietary components, vitamin C and zinc intake were found to have significant associations with DepS. Specifically, patients with DepS exhibited lower intakes of dietary vitamin C and manganese compared with those without DepS. In multivariable analyses, vitamin C showed a significant linear association with DepS, whereas zinc demonstrated a significant non-linear association. Clinical studies have shown that higher vitamin C levels are associated with improved mood states in young males [57], and vitamin C may contribute to neurotransmitter synthesis and antioxidant defense, which could be relevant in breast cancer patients undergoing cytotoxic treatment [58]. In our study, vitamin C intake demonstrated a linear inverse relationship with DepS, with increasing intake being associated with lower odds of DepS, likely due to its antioxidant properties and involvement in neurotransmitter-related pathways. Zinc displayed a significant non-linear association with DepS across all models. At lower levels of zinc intake, increasing intake was associated with lower odds of DepS. However, this inverse association appeared to weaken as intake increased. Because confidence intervals widened and the number of observations decreased at the upper end of the zinc distribution, estimates in this range should be interpreted cautiously. Zinc deficiency, which can occur due to insufficient dietary intake, has been linked to DepS in both animal and human studies [59,60]. A meta-analysis reported that higher zinc status was associated with reduced depression risk [61]. Zinc also modulates synaptic transmission in the hippocampus and amygdala [62]. As an important cofactor for SOD, inadequate zinc intake may impair endogenous antioxidant defense capacity, potentially contributing to oxidative stress-related DepS [63]. Additionally, although *n*-3 PUFAs and folate differed between participants with and without DepS in univariate analyses, these associations were no longer statistically significant after adjustment for potential confounders, suggesting the observed relationships may be explained by demographic, clinical, or lifestyle characteristics.

Although no statistically significant associations were observed for vitamins A and E, manganese, and selenium in our study, previous population-based research has reported inverse associations between these nutrients and DepS. For example, the ELSA-Brasil cohort study observed significant associations between higher dietary intakes of these nutrients and a reduced risk of depression [64]. The discrepancy between studies may arise from differences in study populations, sample sizes, dietary assessment methods, and depression measurement tools. Notably, the ELSA-Brasil study was conducted in a general adult population, whereas our study focused specifically on breast cancer patients undergoing chemotherapy, who may experience unique metabolic and psychological stressors that influence nutrient-mood relationships. Although the associations in our study were not statistically significant after adjustment, the overall evidence suggests that adequate antioxidant intake may be relevant to psychological health, particularly in vulnerable populations such as cancer patients. These findings highlight the potential importance of considering dietary factors in the management of DepS among breast cancer patients. Healthcare providers may consider offering general nutritional guidance that encourages balanced diets rich in fruits, vegetables, and other antioxidant-containing foods as part of comprehensive supportive care.

To our knowledge, this is among the first studies to investigate the association between CDAI and DepS in breast cancer patients undergoing chemotherapy. Unlike prior studies conducted in general or metabolically at-risk populations, our study focused on a clinically distinct group characterized by substantial oxidative stress related to both the disease and its treatment. Furthermore, by incorporating oxidative stress biomarkers into the analytical framework, this study extends previous research by examining whether these biomarkers statistically account for part of the observed association between CDAI and DepS. Although causal inferences cannot be drawn from this cross-sectional design, our findings provide a more integrated perspective on the relationships among dietary antioxidant intake, oxidative stress status, and DepS in breast cancer patients.

This study has several limitations that should be acknowledged. First, CDAI was calculated based on a limited set of antioxidant nutrients and may not comprehensively reflect the overall dietary antioxidant capacity, as other bioactive compounds, such as polyphenols and carotenoids, were not included. Second, given the cross-sectional design, the temporal relationship between dietary antioxidant intake and DepS cannot be established, and the possibility of reverse causation cannot be excluded. Third, dietary intake was assessed using three non-consecutive 24 h dietary recalls based on self-report, which may be subject to recall bias and measurement error. Furthermore, CDAI was calculated from dietary recall data and food composition tables rather than direct biochemical measurements. The actual antioxidant micronutrient content of foods may vary according to factors such as geographical origin, soil composition, agricultural practices, seasonality, storage conditions, and food processing. Therefore, the calculated CDAI may not fully reflect individuals’ actual antioxidant micronutrient intake. Fourth, although multiple potential confounders were adjusted for, residual confounding from unmeasured factors may still exist. Fifth, the relatively modest sample size may have limited statistical power and restricted further subgroup analyses. In addition, only 113 of 302 participants provided blood samples for oxidative stress biomarker assessment, which may have introduced selection bias and limited the representativeness of biomarker-related analyses. Although IPW sensitivity analyses yielded results consistent with the primary findings, residual selection effects cannot be completely excluded. Furthermore, oxidative stress biomarkers measured were measured in peripheral blood and may not fully reflect oxidative processes occurring in specific tissues or organs. Therefore, the findings should be interpreted with caution. Future multicenter prospective studies with larger sample sizes are needed to validate these findings and further clarify the relationships among dietary antioxidant intake, oxidative stress status, and DepS among patients with breast cancer.

## 5. Conclusions

In conclusion, this study found a significant inverse association between CDAI and DepS among patients with breast cancer. However, analyses of the individual CDAI components suggested that this association may be primarily driven by selected antioxidant nutrients, particularly vitamin C, while zinc showed a significant non-linear association. Plasma SOD and GSH statistically accounted for part of the observed association, suggesting a potential link between dietary antioxidant intake, oxidative stress status, and DepS. Given the cross-sectional design, these findings should be interpreted as associations rather than evidence of causality. Nevertheless, the results provide preliminary evidence supporting further investigation of the relationships among dietary antioxidant intake, oxidative stress and psychological health among breast cancer patients. Further prospective and interventional studies are warranted to confirm these findings and clarify the respective contributions of individual antioxidant nutrients and the underlying biological mechanisms.

## Figures and Tables

**Table 1:** Characteristics of breast cancer patients with and without DepS (*n* = 302).

Variables	Without DepS (*n* = 200)	With DepS (*n* = 102)	t/z/χ^2^	*p*
Age (y) ^a^	52.62 ± 11.18	54.51 ± 11.13	−1.395	0.164
BMI (kg/m^2^) ^b^	23.9 (22.1, 25.7)	24.3 (22.2, 26.4)	−1.276	0.202
Marital status, *n* (%) ^d^				
Widowed/divorced/single	6 (3.0)	5 (4.9)	0.696	0.404
Married	194 (97.0)	97 (95.1)		
Education level, *n* (%) ^b^				
Primary school or lower	37 (18.5)	31 (30.4)	8.269	0.041
Middle school	60 (30.0)	33 (32.4)		
High school/secondary school	44 (22.0)	20 (19.6)		
Junior college or higher	59 (29.5)	18 (17.6)		
Employment, *n* (%) ^c^				
Employed	67 (33.5)	19 (18.6)	3.384	0.184
Unemployed	38 (19.0)	25 (24.5)		
Retirement	95 (47.5)	58 (56.9)		
Residence, *n* (%) ^c^				
Rural areas	48 (24.0)	36 (35.3)	5.800	0.055
Towns	16 (8.0)	11 (10.8)		
Urban areas	136 (68.0)	55 (53.9)		
Family monthly income (CNY), *n* (%) ^c^				
<3000	13 (6.5)	17 (16.7)	8.175	0.017
3000~5000	87 (43.5)	43 (42.2)		
>5000	100 (50.0)	42 (41.1)		
Physical activity level, *n* (%) ^c^				
Low	44 (22.0)	29 (28.4)	1.792	0.408
Moderate	147 (73.5)	70 (68.6)		
High	9 (4.5)	3 (3.0)		
Menopausal status, *n* (%) ^c^				
Post-menopausal	119 (59.5)	65 (63.7)	0.507	0.477
Pre-menopausal	81 (40.5)	37 (36.3)		
Pain VAS score ^b^	0.0 (0.0, 1.0)	0.0 (0.0, 1.0)	−1.080	0.280
PSQI score ^b^	5.0 (3.0, 7.0)	6.0 (4.0, 9.0)	−2.953	0.003
Chemotherapy cycle, *n* (%) ^c^				
T0	94 (47.0)	57 (55.9)	2.181	0.536
T1~T2	70 (35.0)	30 (29.4)		
T3~T4	18 (9.0)	8 (7.8)		
≥T5	18 (9.0)	7 (6.9)		
Cancer stage, *n* (%) ^c^				
I	64 (32.0)	34 (33.3)	0.055	0.973
II	116 (58.0)	58 (56.9)		
III	20 (10.0)	10 (9.8)		
Surgery type, *n* (%) ^c^				
Lumpectomy	72 (36.0)	41 (40.2)	0.508	0.476
Mastectomy	128 (64.0)	61 (59.8)		

Data are shown as n (%), median (25th and 75th percentiles), or mean ± standard deviation. DepS, depressive symptoms; BMI, body mass index; CNY, Chinese yuan; VAS, Visual Analog Scale; PSQI, Pittsburgh Sleep Quality Index. ^a^ Independent samples *t*-test; ^b^ Mann–Whitney U test; ^c^ Chi-squared test; ^d^ Chi-squared test with continuity correction.

**Table 2:** CDAI scores and dietary nutrient intakes in breast cancer patients with and without DepS (*n* = 302).

Variables	Without DepS (*n* = 200)	With DepS (*n* = 102)	t/z/χ^2^	*p*
CDAI (continuous)	0.3 (−1.6, 2.2)	−2.4 (−3.5, −0.21)	−6.310	<0.001
Nutrients				
Vitamin A (µgRAE/d)	409.0 (317.3, 585.5)	398.5 (283.8, 574.3)	−0.669	0.503
Vitamin C (mg/d)	120.0 (80.1, 183.4)	78.1 (41.9, 117.4)	−5.830	<0.001
Vitamin E (mg/d)	16.1 (11.7, 20.0)	14.7 (10.4, 18.6)	−1.868	0.062
Zinc (mg/d)	9.3 (7.3, 11.4)	9.0 (5.8, 12.5)	−1.197	0.231
Manganese (mg/d)	3.6 (2.7, 5.2)	3.0 (2.3, 4.0)	−3.330	0.001
Selenium (µg/d)	50.5 (34.1, 73.8)	47.0 (29.2, 69.1)	−1.232	0.218
*n*-3 PUFAs (g/day)	1.1 (0.8, 1.6)	1.0 (0.6, 1.5)	−1.981	0.048
Folate (µg/day)	110.0 (69.1, 157.3)	87.0 (53.2, 145.2)	−2.384	0.017

Data are shown as median (25th and 75th percentiles). DepS, depressive symptoms; CDAI, Composite Dietary Antioxidant Index; *n*-3 PUFAs, *n*-3 polyunsaturated fatty acids. Mann–Whitney U test was used.

**Table 3:** Comparison of linear and non-linear associations between CDAI and depression symptoms.

Variable	Model	LRT (*p*)	AIC	BIC
CDAI	Linear	—	353.98	391.09
	Nonlinear	0.024	350.53	395.06
Vitamin A	Linear	—	383.35	420.46
	Nonlinear	0.153	383.60	428.13
Vitamin C	Linear	—	354.68	391.79
	Nonlinear	0.321	356.41	400.93
Vitamin E	Linear	—	383.14	420.24
	Nonlinear	0.684	386.38	430.90
Zinc	Linear	—	382.40	419.50
	Nonlinear	<0.001	362.33	406.86
Manganese	Linear	—	380.33	420.45
	Nonlinear	0.249	381.55	429.82
Selenium	Linear	—	383.34	417.43
	Nonlinear	0.360	385.30	426.07

CDAI, Composite Dietary Antioxidant Index; LRT, likelihood ratio test; AIC, Akaike Information Criterion; BIC, Bayesian Information Criterion. The likelihood ratio test was used to compare the linear model with the restricted cubic spline (RCS) model, both adjusted for age, BMI, education level, family monthly income, cancer stage, chemotherapy cycle, PSQI score, and total energy intake. Lower AIC and BIC values indicate better model fit.

**Table 4:** Associations between linear CDAI components and depressive symptoms in breast cancer patients (*n* = 302).

Variables	OR	95% CI	*p*
Vitamin A	1.000	0.999, 1.001	0.963
Vitamin C	0.989	0.984, 0.993	<0.001
Vitamin E	0.991	0.953, 1.028	0.642
Selenium	1.000	0.993, 1.007	0.915
Manganese	0.943	0.844, 1.005	0.201

OR, odds ratio; CI, confidence interval. Logistic regression analysis was fully adjusted for age, BMI, education level, family monthly income, cancer stage, chemotherapy cycle, PSQI score and total energy intake.

**Table 5:** Association between CDAI and plasma oxidative stress biomarkers in patients with breast cancer (*n* = 113).

Variables	β	95% CI	*p*
SOD	0.750	0.573, 0.975	<0.001
GSH	0.615	0.479, 0.771	<0.001

Multivariable linear regression was performed after adjusting for age, BMI, education level, family monthly income, cancer stage, chemotherapy cycle, PSQI score, and total energy intake. 95% CI, 95% confidence interval; CDAI, Composite Dietary Antioxidant Index; SOD, superoxide dismutase; GSH, glutathione.

**Table 6:** Association between plasma oxidative stress biomarkers and depressive symptoms in patients with breast cancer (*n* = 113).

Variables	β	95% CI	*p*
SOD	−0.610	−0.732, −0.489	<0.001
GSH	−0.900	−1.045, −0.756	<0.001

Multivariable linear regression was performed after adjusting for age, BMI, education level, family monthly income, cancer stage, chemotherapy cycle, PSQI score, and total energy intake. 95% CI, 95% confidence interval; SOD, superoxide dismutase; GSH, glutathione.

**Table 7:** Exploratory Mediation analysis of oxidative stress biomarkers in the association between CDAI and depressive symptoms in patients with breast cancer (*n* = 113).

Mediators	ATE			ADE			ACME		
	Estimate	95% CI	*p*	Estimate	95% CI	*p*	Estimate	95% CI	*p*
GSH	−0.918	−1.081, −0.809	<0.001	−0.726	−0.909, −0.564	<0.001	−0.192	−0.338, −0.103	<0.001
SOD	−0.918	−1.081, −0.809	<0.001	−0.789	−0.985, −0.648	<0.001	−0.129	−0.228, −0.050	<0.001

Mediation analysis was performed after adjusting for age, BMI, education level, family monthly income, cancer stage, chemotherapy cycle, PSQI score, and total energy intake. The 95% CI was computed from 5000 non-parametric bootstrap replicates. ATE, average total effect; ADE, average direct effect; ACME, average causal mediation effect; 95% CI, 95% confidence interval; CDAI, Composite Dietary Antioxidant Index; SOD, superoxide dismutase; GSH, glutathione.

## Data Availability

Data are available upon reasonable request from the corresponding author. The data contain information from human participants, and access is restricted to protect participant privacy and comply with ethical regulations.

## Supplementary Materials

The following supporting information can be downloaded at https://www.mdpi.com/article/10.3390/nu18142230/s1: Figure S1: Path diagram of the mediation analysis of oxidative stress biomarkers on the association between CDAI and depressive symptoms in patients with breast cancer; Table S1: Associations between CDAI components intake and depressive symptoms in breast cancer patients; Table S2: Comparison of linear and non-linear associations of dietary *n*-3 polyunsaturated fatty acids and folate intake with depressive symptoms in breast cancer patients; Table S3: Associations of dietary folate and *n*-3 polyunsaturated fatty acids intake with depressive symptoms in breast cancer patients; Table S4: Comparison of characteristics between participants with and without plasma oxidative stress biomarkers; Table S5: Inverse probability weighting (IPW) sensitivity analyses for the biomarker subgroup; Table S6: Model diagnostics for the association between CDAI and oxidative stress biomarkers; Table S7: Model diagnostics for the association between oxidative stress biomarkers and depressive symptoms.

## Abbreviations

The following abbreviations are used in this manuscript:

ACME	Average Causal Mediation Effect
ADE	Average Direct Effect
AIC	Akaike Information Criterion
ATE	Average Total Effect
BIC	Bayesian Information Criterion
BMI	Body Mass Index
CAT	Catalase
CDAI	Composite Dietary Antioxidant Index
CI	Confidence Intervals
CNY	Chinese Yuan
DepS	Depressive Symptoms
GSH	Glutathione
GSH-Px	Glutathione Peroxidase
HADS-D	Hospital Anxiety and Depression Scale—Depression subscale
IPAQ-SF	International Physical Activity Questionnaire
IPW	Inverse Probability Weighting
MDA	Malondialdehyde
MET	Metabolic Equivalent of Task
MnSOD	Mitochondrial Superoxide Dismutase
ORs	Odds Ratios
PSQI	Pittsburgh Sleep Quality Index
PUFAs	Polyunsaturated Fatty Acids
RCS	Restricted Cubic Spline
ROS	Reactive Oxygen Species
SD	Sleep Disturbance
SOD	Superoxide Dismutase
VAS	Visual Analogue Scale
