# Supplementary material for: Association Between Composite Dietary Antioxidant Index and Depressive Symptoms in Breast Cancer Patients: The Role of Oxidative Stress Biomarkers in a Cross-Sectional Study
Source: Nutrients. 2026 Jul 9;18(14):2230. doi: 10.3390/nu18142230 (PMC13414930; doi:10.3390/nu18142230)
Supplement: Supplementary file 1 [file nutrients-18-02230-s001.zip › nutrients-4345405-supplementary.pdf]

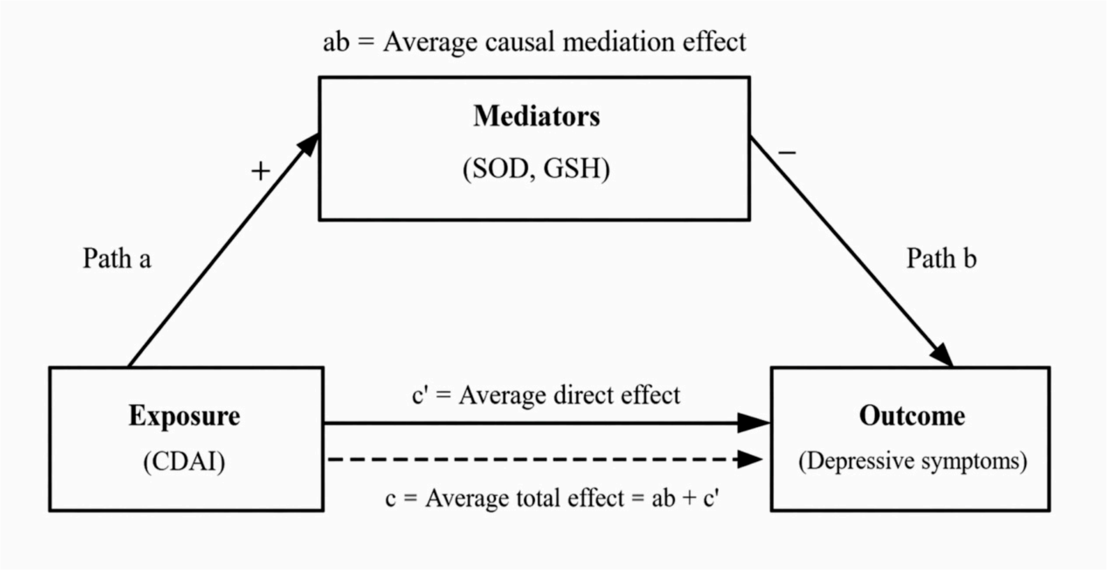

Figure S1. Path diagram of the mediation analysis of oxidative stress biomarkers on the association between CDAI and depressive symptoms in patients with breast cancer. SOD, superoxide dismutase; GSH, glutathione; CDAI, Composite Dietary Antioxidant Index.

Table S1. Associations between CDAI components intake and depressive symptoms in breast cancer patients (*n* = 302).

Nutrient	Model 1			Model 2		
	**OR**	**95% CI**	***P***	**OR**	**95% CI**	***P***
Vitamin A	1.000	0.999, 1.001	0.527	1.000	0.999, 1.001	0.610
Vitamin C	0.988	0.984, 0.993	<0.001	0.988	0.984, 0.993	<0.001
Vitamin E	0.969	0.937, 0.999	0.049	0.968	0.936, 0.998	0.046
Selenium	0.997	0.991, 1.004	0.425	0.998	0.991, 1.004	0.443
Manganese	0.901	0.801, 0.986	0.056	0.905	0.805, 0.990	0.068

CDAI, Composite Dietary Antioxidant Index; OR, odds ratio; CI, confidence interval. Model 1, unadjusted; Model 2, adjusted for age and BMI.

Table S2. Comparison of linear and non-linear associations of dietary n-3 polyunsaturated fatty acids and folate intake with depressive symptoms in breast cancer patients (*n* = 302).

Variable	Model	LRT (*P*)	AIC	BIC
n-3 PUFAs	Linear	—	390.76	450.13
	Non-linear	0.062	389.20	455.99
Folate	Linear	—	391.10	450.47
	Non-linear	0.173	391.60	458.39

PUFAs, polyunsaturated fatty acids; AIC, Akaike Information Criterion; BIC, Bayesian Information Criterion. The likelihood ratio test, adjusted for age, BMI, education level, family monthly income, cancer stage, chemotherapy cycle, PSQI scores and total energy intake, was used to compare the linear model with the restricted cubic spline model. Lower AIC and BIC values indicate better model fit.

Table S3. Associations of dietary folate and n-3 polyunsaturated fatty acids intake with depressive symptoms in breast cancer patients (*n* = 302).

Nutrient	Model 1			Model 2			Model 3		
	**OR**	**95% CI**	***P***	**OR**	**95% CI**	***P***	**OR**	**95% CI**	***P***
n-3 PUFAs	0.755	0.552, 1.066	0.122	0.740	0.508, 1.048	0.100	0.886	0.577, 1.332	0.568
Folate	0.998	0.996, 1.001	0.219	0.998	0.996, 1.001	0.209	0.999	0.997, 1.002	0.643

PUFAs, polyunsaturated fatty acids; OR, odds ratio; CI, confidence interval. Data are presented as odds ratios with 95% confidence intervals and *P* values. Logistic regression analyses were performed. Model 1, unadjusted; Model 2, adjusted for age and BMI; Model 3, adjusted for age, BMI, education level, family monthly income, chemotherapy cycle, cancer stage, PSQI score, and total energy intake.

Table S4. Comparison of characteristics between participants with and without plasma oxidative stress biomarkers (*n* = 302).

Variables	Without oxidative stress biomarkers (n = 189)	With oxidative stress biomarkers (n = 113)	$t/z/\chi^2$	*P*
CDAI (continuous)[a]	−0.28 (−2.33, 1.76)	−0.60 (−2.94, 1.82)	−0.396	0.692
Age (y)[a]	54 (47, 60)	52 (45, 61)	−0.819	0.413
BMI (kg/m^2)[a]	24.0 (21.9, 25.9)	24.1 (22.3, 26.2)	−0.667	0.499
Marital status, n (%)[d]				
Widowed/divorced/single	8 (4.2)	3 (2.7)	0.502	0.479
Married	181 (95.8)	110 (97.3)		
Education level, n (%)[b]				
Primary school or lower	42 (22.2)	25 (22.1)	2.005	0.571
Middle school	60 (31.7)	39 (34.5)		
High school/secondary school	44 (23.3)	19 (16.8)		
Junior college or higher	43 (22.8)	30 (26.6)		
Employment, n (%)[c]				
Employed	47 (24.9)	37 (32.7)	4.407	0.110
Unemployed	45 (23.8)	17 (15.0)		
Retirement	97 (51.3)	59 (52.3)		
Residence, n (%)[c]				
Rural areas	38 (20.1)	31 (27.4)	3.341	0.188
Towns	18 (9.5)	6 (5.3)		
Urban areas	133 (70.4)	76 (67.3)		
Family monthly income (CNY), n (%)[c]				
< 3000	19 (10.1)	11 (9.7)	3.787	0.151
3000 ~ 5000	89 (47.1)	41 (36.3)		
> 5000	81 (42.8)	61 (54.0)		
Physical activity level, n (%)[c]				
Low	52 (27.5)	21 (18.6)	3.598	0.165
Moderate	131 (69.3)	86 (76.1)		
High	6 (3.2)	6 (5.3)		
Menopausal status, n (%)[c]				
Post-menopausal	118 (62.4)	66 (58.4)	0.482	0.488
Pre-menopausal	71 (37.6)	47 (41.6)		
VAS score[b]	0.0 (0.0, 1.0)	0.0 (0.0, 1.0)	−0.635	0.525
PSQI score[b]	6.0 (3.0, 8.0)	4.0 (3.0, 7.0)	−2.629	0.009
Chemotherapy cycle, n (%)[c]				

Variables	Without oxidative stress biomarkers (n = 189)	With oxidative stress biomarkers (n = 113)	$t/z/\chi^2$	*P*
T0	102 (54.0)	49 (43.3)	7.178	0.066
T1~T2	52 (27.5)	48 (42.5)		
T3~T4	18 (9.5)	8 (7.1)		
≥T5	17 (9.0)	8 (7.1)		
Cancer stage, n (%)[c]				
Ⅰ	66 (34.9)	32 (28.3)	2.016	0.365
Ⅱ	103 (54.5)	71 (62.8)		
Ⅲ	20 (10.6)	10 (8.9)		
Surgery type, n (%)[c]				
Lumpectomy	70 (37.0)	43 (38.1)	0.031	0.860
Mastectomy	119 (63.0)	70 (61.9)		

Data are shown as *n* (%), median (25th and 75th percentiles), or mean ± standard deviation. BMI, body mass index; CNY, China yuan; VAS, visual analog scale; PSQI, Pittsburgh sleep quality index. [a] Independent samples *t*-test. [b] Mann–Whitney U test. [c] Chi-squared test. [d] Chi-squared test with continuity correction.

Table S5. Inverse probability weighting (IPW) sensitivity analyses for the biomarker subgroup (*n* = 113).

Outcome	Exposure	Original β	IPW β (95% CI)	*P* (IPW)
SOD	CDAI	0.750	0.715 (0.537, 0.894)	< 0.001
GSH	CDAI	0.615	0.632 (0.505, 0.758)	< 0.001
HADS-D	SOD	–0.610	–0.647 (–0.803, –0.492)	< 0.001
HADS-D	GSH	–0.900	–0.848 (–1.019, –0.678)	< 0.001

Inverse probability weighting (IPW) was applied to evaluate potential selection bias due to blood sample availability. Original β values represent estimates from conventional multivariable linear regression models, while IPW β values represent weighted estimates. All models were adjusted for age, BMI, education level, family monthly income, chemotherapy cycle, cancer stage, PSQI score, and total energy intake. SOD, superoxide dismutase; GSH, glutathione; CDAI, composite dietary antioxidant index; HADS-D, depression subscale of the Hospital Anxiety and Depression Scale.

Table S6. Model diagnostics for the association between CDAI and oxidative stress biomarkers.

Variables	R^2	Max VIF	Normality (*P*)	Homoscedasticity (*P*)
SOD	0.441	1.38	< 0.001	0.288
GSH	0.530	1.38	< 0.001	0.513

All models were adjusted for age, BMI, education level, family monthly income, chemotherapy cycle, cancer stage, PSQI score, and total energy intake. A maximum Variance Inflation Factor (VIF) < 5 indicates no substantial multicollinearity among covariates. Residual normality was assessed using the Shapiro–Wilk test. For models with non-normal residuals (Normality *P* < 0.05), non-parametric bootstrapping was performed to obtain robust estimates and confidence intervals. SOD, superoxide dismutase; GSH, glutathione; CDAI, composite dietary antioxidant index.

Table S7. Model diagnostics for the association between oxidative stress biomarkers and depressive symptoms.

Variables	R^2	Max VIF	Normality (*P*)	Homoscedasticity (*P*)
SOD	0.537	1.38	0.199	0.011
GSH	0.636	1.38	< 0.001	0.153

All models were adjusted for age, BMI, education level, family monthly income, chemotherapy cycle, cancer stage, PSQI score, and total energy intake. A maximum Variance Inflation Factor (VIF) < 5 indicates no substantial multicollinearity among covariates. Residual normality was assessed using the Shapiro–Wilk test. For models with non-normal residuals (Normality $P < 0.05$), non-parametric bootstrapping was performed to obtain robust estimates and confidence intervals. SOD, superoxide dismutase; GSH, glutathione.
